# A glimpse into the future: revealing the key factors for survival in cognitively impaired patients

**DOI:** 10.3389/fnagi.2024.1376693

**Published:** 2024-07-04

**Authors:** Libing Wei, Dikang Pan, Sensen Wu, Hui Wang, Jingyu Wang, Lianrui Guo, Yongquan Gu

**Affiliations:** ^1^Xuanwu Hospital, Capital Medical University, Beijing, China; ^2^Renal Division, Peking University First Hospital, Beijing, China

**Keywords:** cognitive impairment, NHANES, mortality, nomogram, prospective studies

## Abstract

**Background:**

Drawing on prospective data from the National Health and Nutrition Examination Survey (NHANES), our goal was to construct and validate a 5-year survival prediction model for individuals with cognitive impairment (CI).

**Methods:**

This study entailed a prospective cohort design utilizing information from the 2011–2014 NHANES dataset, encompassing individuals aged 40 years or older, with updated mortality status as of December 31, 2019. Predictive models within the derivation and validation cohorts were assessed using logistic proportional risk regression, column-line plots, and least absolute shrinkage and selection operator (LASSO) binomial regression models.

**Results:**

The study enrolled a total of 1,439 participants (677 men, mean age 69.75 ± 6.71 years), with the derivation and validation cohorts consisting of 1,007 (538 men) and 432 (239 men) individuals, respectively. The 5-year mortality rate stood at 16.12% (*n* = 232). We devised a 5-item column-line graphical model incorporating age, race, stroke, cardiovascular disease (CVD), and blood urea nitrogen (BUN). The model exhibited an area under the curve (AUC) of 0.772 with satisfactory calibration. Internal validation demonstrated that the column-line graph model displayed strong discrimination, yielding an AUC of 0.733, and exhibited good calibration.

**Conclusion:**

To sum up, our study successfully developed and internally validated a 5-item nomogram integrating age, race, stroke, cardiovascular disease, and blood urea nitrogen. This nomogram exhibited robust predictive performance for 5-year mortality in individuals with CI, offering a valuable tool for prognostic evaluation and personalized care planning.

## Introduction

Cognitive functioning encompasses a wide array of functions, spanning verbal and non-verbal memory, attention, executive functioning, language, and motor skills, where impairments often manifest across multiple cognitive domains, affecting language, computation, judgment, memory, and executive functioning. These deficits can lead to behavioral, emotional, and personality abnormalities, ultimately diminishing work capacity and daily task performance, imposing significant financial and psychological strains on families and society (Gavelin et al., 2021; Chehrehnegar et al., 2022; Huang et al., 2022).

The repercussions of cognitive impairment (CI) extend beyond individual health, impacting independence, productivity, and necessitating substantial social, medical, and financial resources. With increasing life expectancies, age-related cognitive decline emerges as a pressing challenge for older individuals globally, underscoring the critical public health importance of cognitive well-being (Hebert et al., 2013; Knapp and Wong, 2020). The early identification of high-risk individuals and timely interventions are pivotal in mitigating premature mortality among older persons with cognitive impairment. Therefore, the imperative lies in the development of mortality prediction models tailored to this demographic, an area where existing studies have been hindered by limited sample sizes, follow-up durations, and generalizability issues to the broader population of cognitively impaired individuals. Notably, a dearth of population-based research exists in constructing mortality risk prediction models for older individuals with cognitive impairment (Xing et al., 2023). Nomogram is a visual statistical prognostic tool that is widely used in clinical prognostic evaluation by calculating scores for potential predictors (Hu et al., 2021).

**Figure 1:**
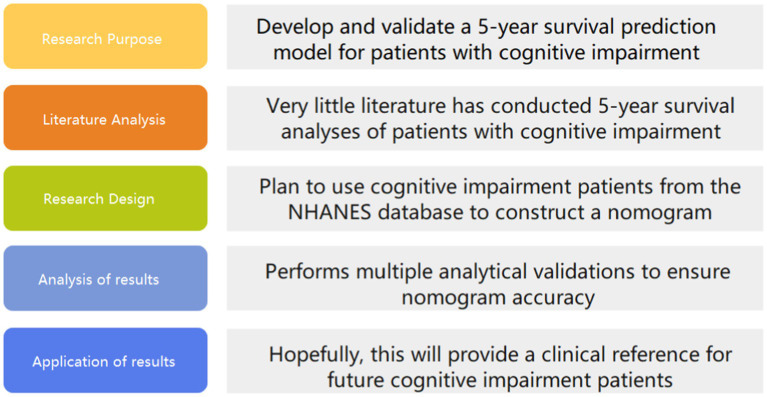
Framework diagram of the study.

This study aims to establish and validate a 5-year all-cause mortality prediction map for older individuals with CI based on a nationally representative U.S. population, furnishing a valuable reference for averting and managing adverse outcomes in this demographic. Moreover, to enhance the elucidation of the objectives and theoretical model outlined in this paper, a conceptual framework (Figure 1) was developed. This framework serves to visually represent the relationships and key components under investigation, providing a structured overview of the study’s aims and the hypothesized model being explored.

## Methods

### Study design and population

**Figure 2:**
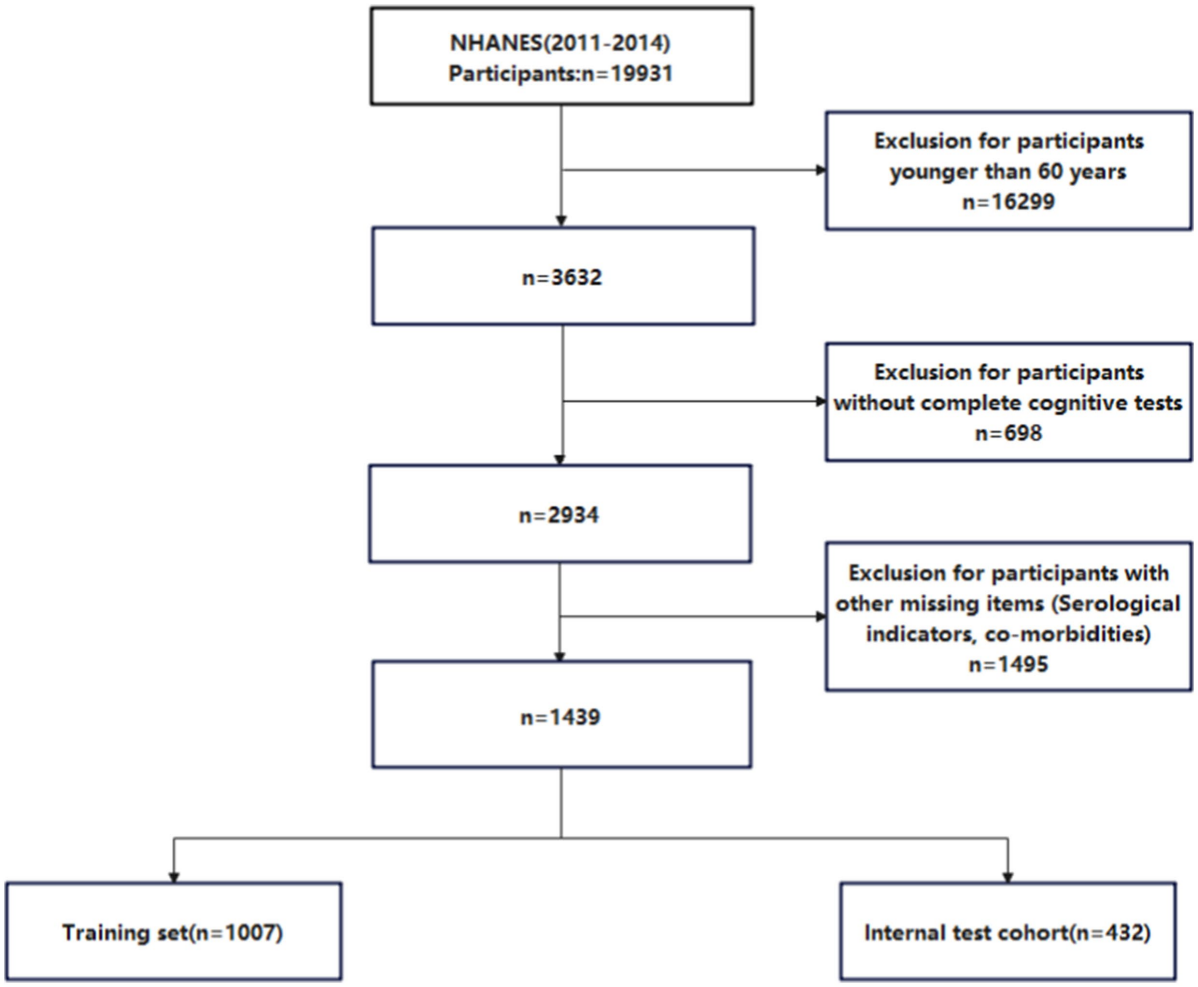
Low chart for participant screening.

NHANES is an ongoing research project that provides estimates of the population’s nutrition and health status in the United States (Pan et al., 2024). It uses a stratified, multi-stage probability design to recruit a representative sample of the American population. Data is collected through structured interviews at home, health screenings at mobile health centers, and laboratory sample analysis (Pan et al., 2023). This study analyzed data from the National Health and Nutrition Examination Survey (NHANES) from 2011 to 2014, which includes information on cognitive function. After screening, a total of 1,439 participants with CI were included. Figure 2 illustrates the flow chart of the study.

### Assessment of cognitive performance

In the 2011–2014 NHANES survey, the assessment of cognitive function in participants aged 60 years or older was conducted through a series of tests. At the end of a private face-to-face interview at a mobile examination center (MEC interview) (Fillenbaum and Mohs, 2023), trained interviewers administered these tests. Three tests were used to evaluate cognitive function: the Consortium to Establish a Registry for Alzheimer’s Disease Word List (CERAD W-L) subtest, which assesses immediate and delayed recall of new verbal information (Desai et al., 2022); the Animal Fluency Test (AFT), which measures categorical verbal fluency (Zhao et al., 2013); and the Digit Symbol Substitution Test (DSST), which evaluates processing speed, sustained attention, and working memory (Liu et al., 2021). These tests have been widely used in mass screening efforts and clinical studies (Walsh et al., 2022; Liang and Zhang, 2024). While cognitive assessments cannot replace a clinical diagnosis based on examination, they are valuable in study cognitive function in relation to various diseases and risk factors.

The CERAD W-L assesses immediate and delayed learning ability for new verbal information. This test involves three consecutive learning trials and a delayed recall test (Luck et al., 2018). During the learning trials, participants are asked to read aloud 10 unrelated words, presented one at a time. They are then asked to recall as many words as possible immediately after each presentation. The order of the words changes in each trial. Participants who cannot read are instructed to repeat each word after it is read out by the interviewer. The delayed recall test takes place after the other cognitive exercises, approximately 8–10 min from the start of the word learning trials (Casagrande et al., 2021).

The AFT assesses categorical verbal fluency, a component of executive function. It requires awareness of animal names, regardless of cultural context, and is not dependent on formal education (Prince et al., 2003). The test has been shown to distinguish between individuals with CI and those with normal cognitive function. Participants are asked to name as many animals as possible in 1 min, and each named animal receives a point (Mcdonnell et al., 2020). NHANES participants first complete a practice test of naming three items of clothing. Those who cannot name three articles of clothing do not proceed with the Animal Fluency Test.

The DSST is a performance module of the Wechsler Adult Intelligence Scale (WAIS III). It is designed to assess processing speed, sustained attention, and working memory (Song et al., 2023). The exercise is conducted using a paper form that has a key at the top containing 9 numbers paired with symbols. Participants are given 2 min to copy the corresponding symbols into the 133 boxes that are adjacent to the numbers (Jiang et al., 2023). The score is calculated based on the total number of correct matches. In NHANES, a sample practice test is administered prior to the main test, allowing participants to familiarize themselves with the task. Participants who were unable to correctly match the symbols with the numbers during the pretest practice were not continued.

In this study, the cognitive scores ranges for CERAD, AFT, and DSST were 0–30, 3–40, and 0–105, respectively. Due to the absence of a gold standard for identifying CI using these three tests, we conducted a comprehensive analysis of previous studies (Timmers et al., 2019; Dong et al., 2020; Shi et al., 2023). To minimize the influence of age on cognitive function, participants were divided into three age groups: 60–69 years, 70–79 years, and 80 years or older. We utilized the lowest quartile of test scores in each group as the threshold for defining cognitive impairment. In these three age groups, the lowest quartiles of CERAD scores were 21, 20, and 17, respectively; for AFT, they were 13, 11, and 11; and for DSST, they were 33, 27, and 25. Therefore, participants with CI were identified based on these criteria.

### Assessment of covariates

Standardized questionnaires were collected on participants’ sociodemographic characteristics, smoking status, diabetes, hypertension, hypercholesterolemia, past diseases and aspirin use. Participants who smoke less than 100 cigarettes in their lifetime are classified as non-smokers, while those who previously smoked more than 100 cigarettes but did not quit are defined as current smokers (Pan et al., 2024). Former smokers were those who used to smoke more than 100 cigarettes but had already quit. Race/ethnicity is classified as Mexican American, other Hispanic, non-Hispanic white, non-Hispanic black, and other races. Education level in our research is classified as lower than high school (less than 9th grade), high school [include 9-12th grade (general educational development or equivalent)], or college or above (some college or Associate’s degree and college graduate or above) (Pan et al., 2023). Marital status is classified into three categories in our researchers, the first being married or living with partners, the second being married, divorced or separated, and the third being unmarried. Poverty income ratio (PIR) scores were defined as less 3, 1–3, and more than 3. It is calculated by dividing the household income by the poverty guidelines of a specific survey year (Song et al., 2023).

### Statistical analysis

All statistical analyses were performed using R software (version 4.3.1). The data collected from the NHANES database were randomly divided into training and validation cohorts at a ratio of 7:3, and the variables were compared. Non-normal data were presented as median (interquartile ranges). For categorical variables, the chi-square test or Fisher’s exact test was used in the univariate analysis, while the *t*-test or rank-sum test was used for continuous variables (Ning et al., 2023). In the training cohort, multivariate analysis was conducted using the least absolute shrinkage and selection operator (LASSO) logistic regression analysis to identify independent risk factors and construct a prediction nomogram for cognitive impairment. The performance of the nomogram was assessed using receiver operating characteristic (ROC) curve and calibration curve. Additionally, a decision curve analysis (DCA) was performed to determine the net benefit threshold of prediction. Results with a *p*-value of <0.05 were considered statistically significant.

## Results

### Baseline characteristics

A total of 1,439 participants were recruited based on predefined criteria, with 1,007 individuals allocated to the development group and 432 to the validation group. Table 1 presents baseline characteristics, including demographics, biochemical indices, co-morbidities, duration of hypertension, and medication use.

**Table 1:** Patient demographics and baseline characteristics.

Characteristic	Cohort		*p*-value
	Training cohort, *N* = 1,007	Internal test cohort, *N* = 432	
Male	538 (53.4%)	239 (55.3%)	0.508
Non-Hispanic White	362 (35.9%)	151 (35.0%)	0.718
Education level			0.305
Less than highschool	393 (39.0%)	182 (42.1%)	
High school	253 (25.1%)	93 (21.5%)	
More than highschool	361 (35.8%)	157 (36.3%)	
Marry status			0.610
Married	532 (52.8%)	217 (50.2%)	
Widowed/Divorced	356 (35.4%)	158 (36.6%)	
Other	119 (11.8%)	57 (13.2%)	
Poverty income ratio			0.986
<1.0	238 (23.6%)	102 (23.6%)	
1.0–3.0	519 (51.5%)	221 (51.2%)	
>3.0	250 (24.8%)	109 (25.2%)	
Smoke			0.775
Never smoker	503 (50.0%)	207 (47.9%)	
Ever smoker	361 (35.8%)	162 (37.5%)	
Current smoker	143 (14.2%)	63 (14.6%)	
Diabetes	308 (30.6%)	123 (28.5%)	0.422
Cardiovascular disease	200 (19.9%)	70 (16.2%)	0.103
Stroke	96 (9.5%)	42 (9.7%)	0.911
Hypertension	657 (65.2%)	280 (64.8%)	0.876
Hypercholesterolaemia	557 (55.3%)	233 (53.9%)	0.630
Depression	860 (85.4%)	367 (85.0%)	0.826
Sleep disorder	106 (10.5%)	50 (11.6%)	0.558
Use of aspirin	812 (80.6%)	347 (80.3%)	0.891
Age	69 (64, 76)	68 (64, 74)	0.062
Albumin	42.0 (40.0, 44.0)	42.0 (40.0, 44.0)	0.297
Alanine aminotransferase	19 (15, 25)	20 (16, 25)	0.194
Aspartate aminotransferase	23 (20, 27)	23 (20, 28)	0.797
Blood urea nitrogen	15 (12, 19)	14 (12, 19)	0.275
Serum Ca	2.35 (2.30, 2.40)	2.35 (2.30, 2.40)	0.548
Creatine Kinase	109 (74, 179)	104 (71, 168)	0.299
Alkaline phosphatase	67 (55, 82)	68 (57, 83)	0.304
Total cholesterol	185 (158, 215)	186 (158, 216)	0.715
Creatinine	86 (72, 103)	81 (68, 102)	0.087
Serum iron	14.0 (10.6, 17.7)	14.3 (10.7, 17.8)	0.678
Serum Phosphorus	1.20 (1.07, 1.32)	1.19 (1.07, 1.30)	0.380
Total bilirubin	10.3 (8.6, 13.7)	11.1 (8.6, 13.7)	0.164
Uric acid	5.60 (4.70, 6.63)	5.50 (4.58, 6.60)	0.153
Triglyceride	129 (89, 183)	121 (82, 179)	0.085
High density lipoprotein	50 (41, 61)	51 (43, 61)	0.163

Frequency (weighted percentages) was presented for categorical variables. Quantitative variables are expressed as median, 25th percentile and 75th percentile.

### Predictive model

**Figure 3:**
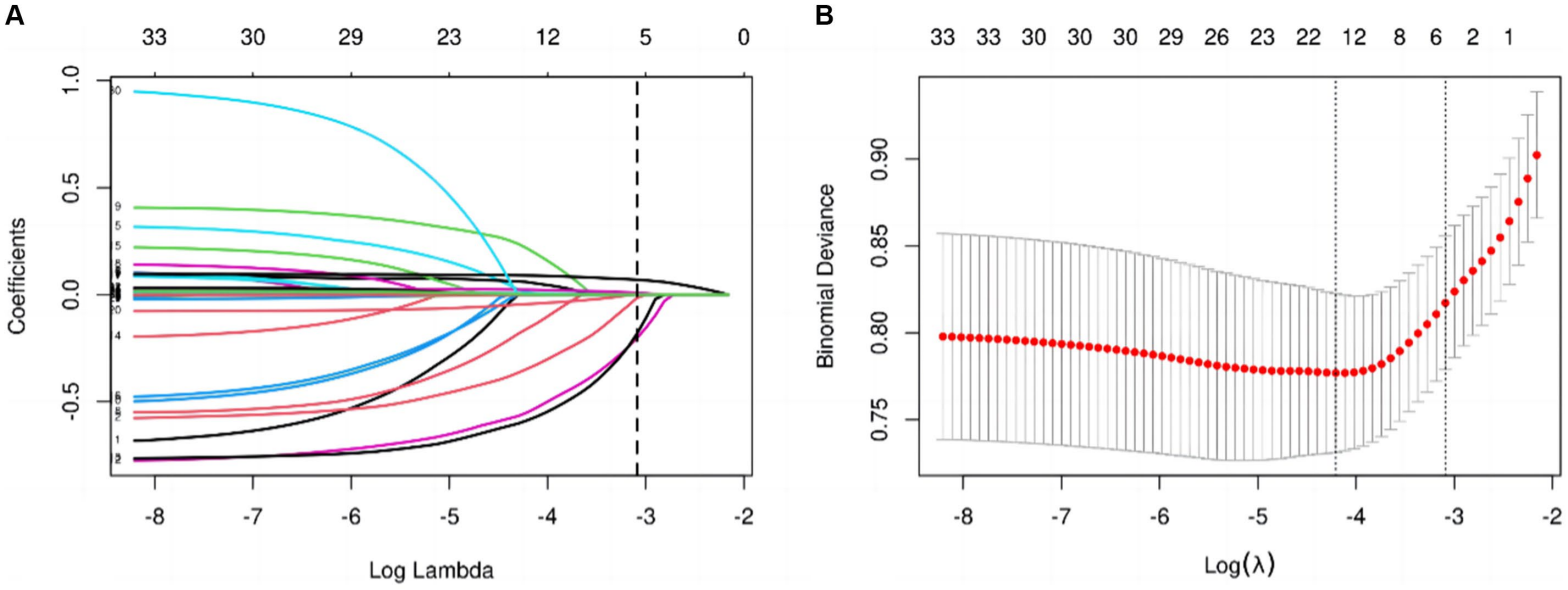
**(A)** LASSO regression coefficient path plot. **(B)** LASSO regression cross-validation plot.

The candidate predictors initially considered in the model encompassed gender, age, race, education level, marital status, smoking status, diabetes, cardiovascular disease, PIR, hyperlipidemia, high-density lipoprotein levels, depression, sleep disorders, stroke, aspirin usage, along with certain hematological indicators. Through LASSO regression analysis conducted in the training cohort, these predictors were streamlined to 5 key variables. The coefficient profile is depicted in Figure 3A, while Figure 3B showcases a cross-validated error plot of the LASSO regression model. The final model, characterized by regularization and parsimony, identified 5 variables as significant predictors: race, cardiovascular disease (CVD), age, stroke, and blood urea nitrogen (BUN). This refined model, exhibiting a cross-validated error within one standard error of the minimum, underscores the predictive strength of these specific variables in the context of the study.

### Development of nomogram

**Figure 4:**
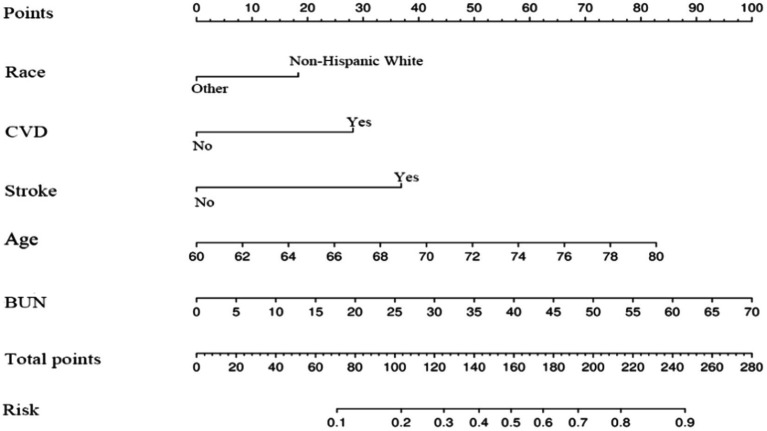
Nomogram prediction model.

The ultimate logistic model integrated 5 independent predictors (race, CVD, age, stroke, BUN) and was transformed into an easily interpretable nomogram, visually depicted in Figure 4. This column chart comprises 8 axes, with axes 2–6 corresponding to each prognostic factor incorporated in the final model. Each predictor is assigned a distinct weighted score within the nomogram. Axes 7 and 8 signify that as the total score increases, the 5-year risk of mortality in individuals with CI escalates accordingly.

### Internal and external validation

**Figure 5:**
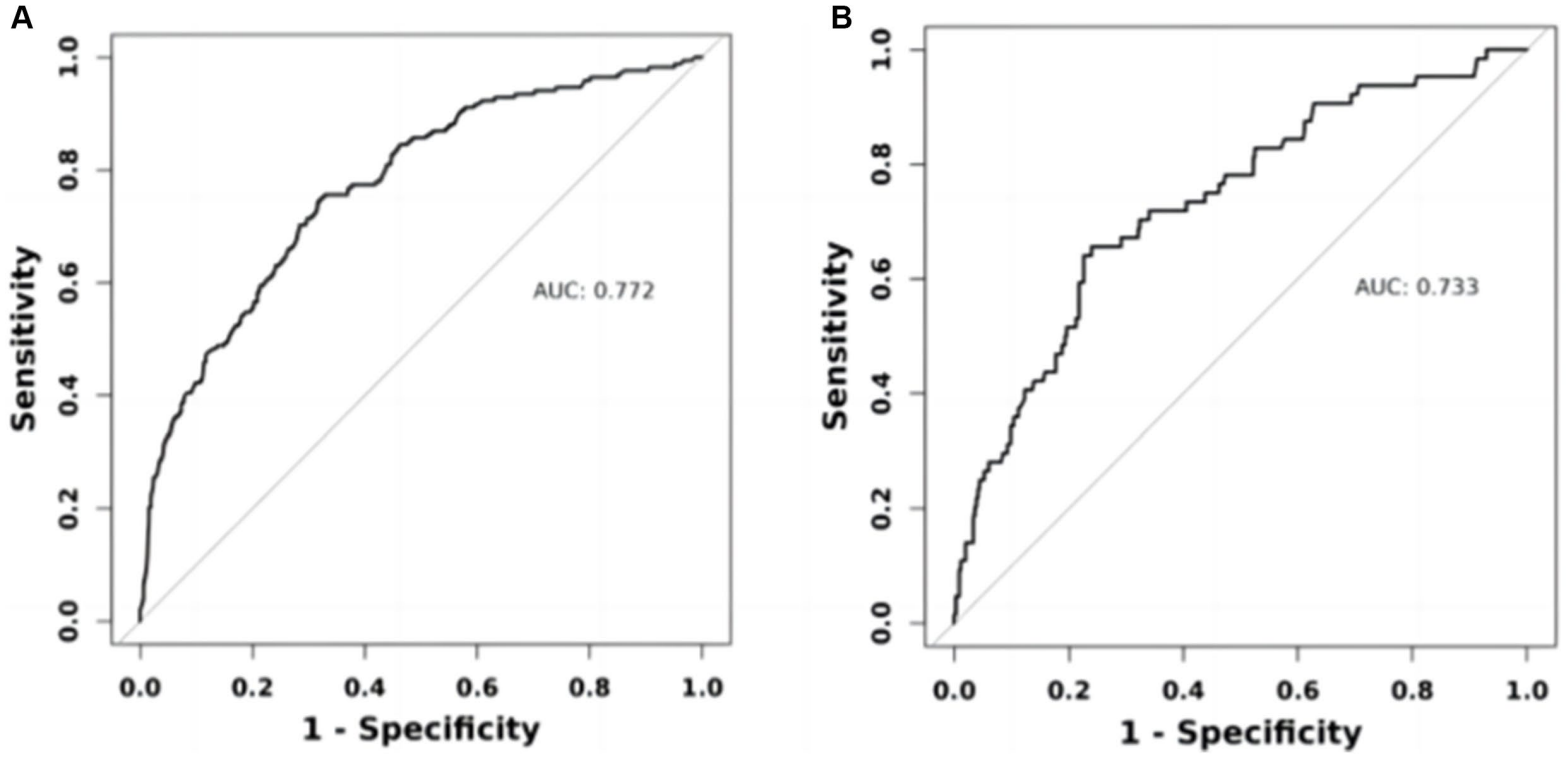
ROC curves of the nomogram prediction model. **(A)** Training set ROC curve. **(B)** Internal validation set ROC curve.

**Figure 6:**
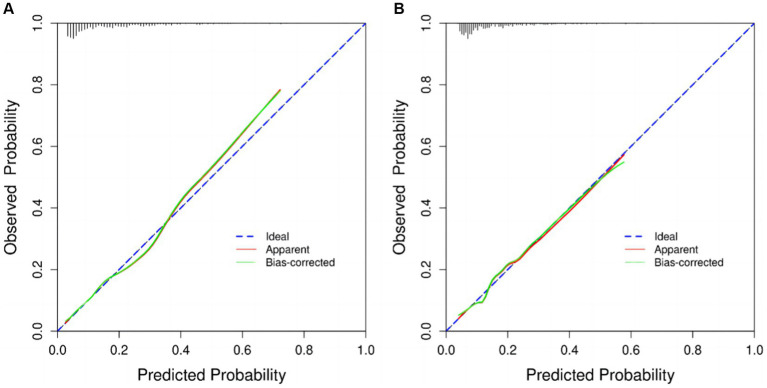
Calibration curves. **(A)** training cohort, **(B)** internal test cohort.

Within the training cohort, the c-index value of 0.772 signifies that the model exhibits commendable discriminative capabilities, as illustrated in Figure 5A. The calibration curves, closely aligned with the diagonal, indicate a high level of concordance between the model’s predictions and the observed outcomes, as depicted in Figure 6A. Transitioning to the internal test cohort, the model maintains its robust discriminative performance, boasting a c-index of 0.733, as showcased in Figure 5B. Furthermore, the calibration plots demonstrate that the model offers a well-fitted representation of CI in the elderly population, as highlighted in Figure 6B.

**Figure 7:**
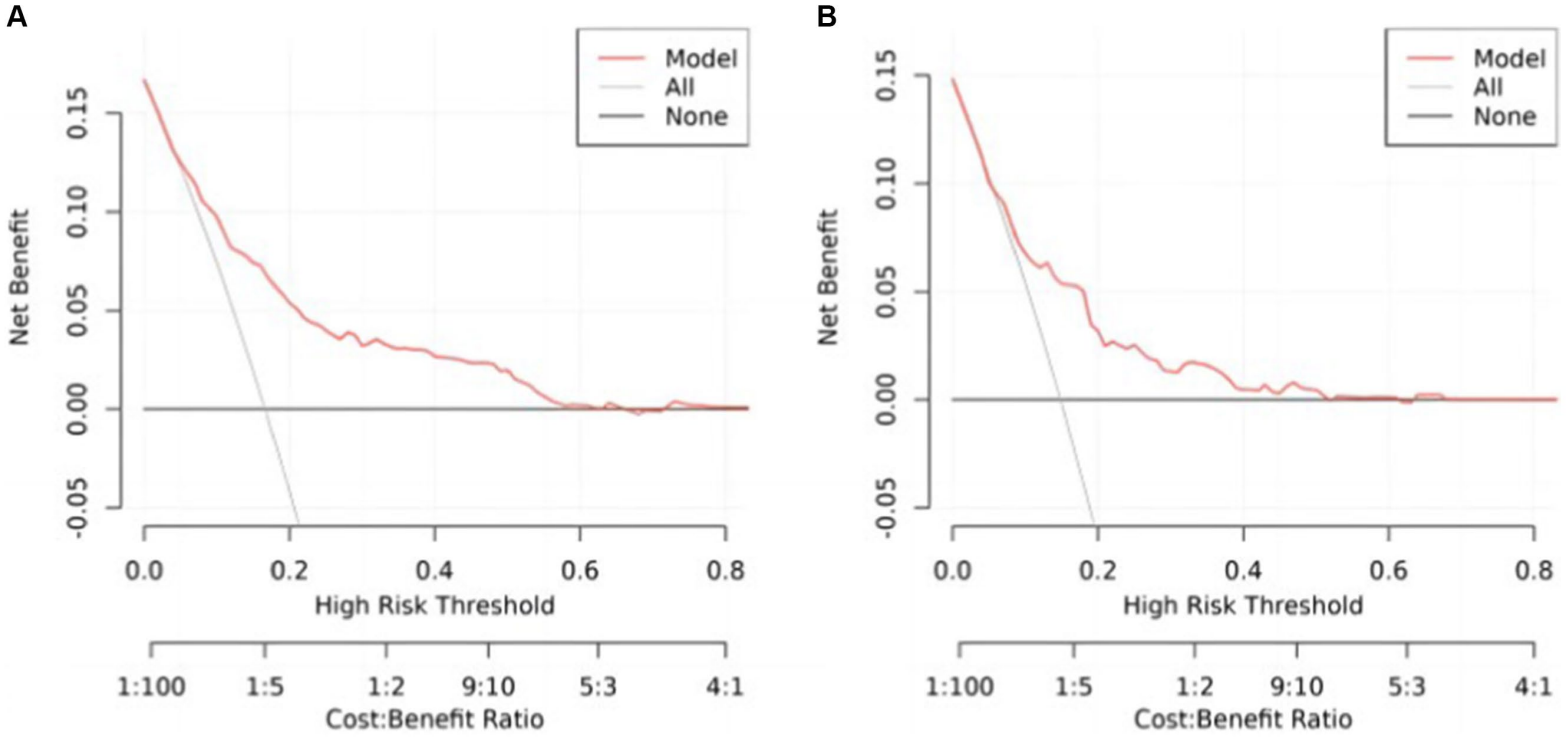
Decision curve analysis, **(A)** training cohort, **(B)** internal test cohort.

The figure below exhibits the DCA curves associated with the nomogram. A high-risk threshold probability signifies the likelihood of notable disparities in the model’s predictions when clinicians confront significant shortcomings while employing the nomogram for diagnostic and decision-making tasks. This study underscores that the nomogram yields considerable net advantages for clinical utilization based on its DCA curve representations (Figures 7A,B).

## Discussion

Our study aimed to construct and validate a predictive model for 5-year mortality risk among patients with CI using data from the NHANES in the United States. The results underscored the importance of developing targeted prognostic tools for this vulnerable population. The model was built on a cohort of 1,439 participants aged 40 and above, revealing a 5-year mortality rate of 16.12% and highlighting significant health risks associated with CI in older adults. Our approach employed logistic proportional hazards regression and innovative techniques such as bar-line plots and LASSO binary regression models to enhance prediction accuracy. Central to our predictive model were five key variables: age, race, history of stroke, CVD, and BUN. Through rigorous statistical analysis, these factors were identified as critical predictors of mortality risk among CI patients. The resulting nomogram visually represents these predictive factors, demonstrating robust performance with an AUC of 0.772, indicating good discriminative ability. Internal validation further confirmed the model’s reliability with an AUC of 0.733 and satisfactory calibration, affirming its applicability in clinical settings. This nomogram not only enhances risk stratification but also facilitates targeted interventions aimed at improving outcomes for individuals with CI, thereby contributing to more effective healthcare management strategies. Future research could further refine the model’s accuracy and explore additional factors to optimize its utility in clinical practice.

In recent years, nomograms have been increasingly used to diagnose and predict a variety of diseases, including cancer (Balachandran et al., 2015), myocardial infarction (Ye et al., 2023), and hypertension (Zhang et al., 2022). The utilization of nomograms serves to simplify the interpretation of pertinent risk factors, aiding both clinicians and patients in navigating the challenges posed by diseases. With the escalating numbers of individuals affected by Alzheimer’s disease and hypertension, the development of a universal risk assessment tool tailored to this demographic becomes increasingly crucial. Despite this pressing need, previous research has not produced analogous nomograms. Hence, our study aimed to establish a prognostic nomogram encompassing demographic characteristics and standard laboratory parameters, furnishing essential insights to shape personalized intervention strategies and forecast 5-year mortality rates in patients with CI. A pivotal outcome of our investigation was the internal validation of our model. We observed that the nomogram exhibited a discriminative power exceeding 0.7 in predicting the five-year mortality rates of CI patients. Moreover, the predicted probabilities closely mirrored the actual probabilities along a 45-degree diagonal, underscoring the robustness and accuracy of our predictive model. These results serve to validate the efficacy and reliability of our prognostic tool.

According to our study, the mortality rate of older CI persons is positively correlated with age, which indicates that the older the age, the higher the mortality rate. Many studies have confirmed the effect of age on cognitive function, and although the mechanisms by which changes in brain structure and function occur with age are not clear, several studies (Schmeidler et al., 2019; Sprague et al., 2019) have shown that various brain functions are affected by a variety of factors, which can lead to cognitive decline. The rate of cognitive decline accelerates with age and can be detected already in middle age (45 to 55 years) (Kohler et al., 2023). In our study, in order to minimise age-related factors, we divided the included population into three different age groups and calculated the lowest quartile of cognitive test scores in each group. However, in our statistical analyses, we found that the effect of age on cognitive functioning could be observed despite the exclusion of some possible biases. Therefore, there is a need for routine screening of cognitive function in the older persons (Travis and Martin, 2020).

There is little research pointing to race on mortality in older CI patients, but studies on Alzheimer’s disease (AD) point to race/ethnicity as a possible influence on life expectancy in AD patients. Schaffert evaluated 1,401 patients with AD, incorporating 21 predictors, and found that white patients with AD possessed less life expectancy (Schaffert et al., 2022). Mehta conducted a retrospective study in which 30,916 AD patients were followed for a mean of 2.4 years and concluded that African American and Latino Alzheimer’s disease patients may have longer survival compared to white Alzheimer’s disease patients (Mehta et al., 2008). The above studies are generally similar to our conclusions, reflecting that more care and attention should be given to the white population.

Several earlier studies have proposed that cardiovascular disease and older CI persons interact with each other and contribute to their respective development. Ren conducted a prospective study aimed at revealing the relevance of CI in patients with heart failure and its prognosis, and found that new-onset dementia was independently associated with an increased risk of all-cause mortality in patients with CVD (Ren et al., 2023). Yono prospectively recruited 585 hypertensive patients, assessed cognitive functioning at baseline, and prospectively identified CVD events, and concluded that the prevalence of cognitive dysfunction was higher in patients with CVD events than in those without (Yano et al., 2014). dysfunction was more prevalent than in patients without CVD events, Van provided insight into the causes of death in dementia patients through a national cohort study (*n* = 59,201), which found that, although the prevalence of CVD was low (18.7% in men and 19.2% in women), it was also one of the leading causes of death in dementia patients (van de Vorst et al., 2016). The above study and our prediction model remind us that patients with CVD combined with CI should be followed up in a targeted manner to prevent bad outcomes.

Stroke is also a risk factor for death in older persons with CI. Rist conducted a cross-sectional study including 10 countries and 6,080 patients and found that approximately 30% of ischaemic stroke survivors exhibited CI and that the risk of dementia was high over time, even in the absence of recurrent strokes, and should therefore be closely monitored for further cognitive decline (Rist et al., 2013). Gale designed a 20-year follow-up study of 921 older persons with CI and found that the relationship between cognitive function and risk of stroke death suggested that cerebrovascular disease is an important cause of cognitive decline (Gale et al., 1996).

Urea nitrogen is one of the main tests reflecting the conditions of renal function, which may be accompanied by renal insufficiency in most patients (Naz and Sulaiman, 2016). And the argument that chronic kidney disease (CKD) is associated with cognitive decline has been supported by some literature. Anatomically, both the kidney and the cranium are low vascular impedance systems that allow continuous, high volume blood perfusion (Tsai et al., 2017), which makes both vulnerable to damage from diseases that cause microvascular damage such as diabetes and hypertension. It has been demonstrated that patients with renal insufficiency are more prone to white matter injury, asymptomatic cerebrovascular haemorrhage and cranial microvascular haemorrhage (Wu et al., 2020). A German prospective study with a 2-year follow-up found an association between moderate - severe renal impairment and dementia (Hiramatsu et al., 2020). To some extent, CKD is closely related to cognitive decline. Meanwhile, BUN is also an easily accessible indicator that can prevent the decline of life expectancy in older persons with CI in advance.

### Limitations

Although our study used a large, representative sample of older Americans, and the NHANES database offers significant advantages in terms of survey methodology and quality control, this article still has some limitations. Firstly, the cohort is based on a population from the U.S. Centers for Disease Control and Prevention, which may not be representative of the wider population, particularly those in low-income countries. Additionally, our model may include potential unmeasured confounders. Future studies should aim to externally validate our nomogram in different populations and settings.

## Conclusion

To sum up, our study successfully developed and internally validated a 5-item nomogram integrating age, race, stroke, cardiovascular disease, and blood urea nitrogen. This nomogram exhibited robust predictive performance for 5-year mortality in individuals with CI, offering a valuable tool for prognostic evaluation and personalized care planning.

## Data availability statement

The raw data supporting the conclusions of this article will be made available by the authors, without undue reservation.

## Ethics statement

The studies involving humans were approved by the National Center for Health Statistics Research Ethics Review Board. The studies were conducted in accordance with the local legislation and institutional requirements. The participants provided their written informed consent to participate in this study.

## Author contributions

LW: Writing – original draft. DP: Writing – original draft. SW: Data curation, Investigation, Writing – review & editing. HW: Data curation, Software, Writing – original draft. JW: Conceptualization, Methodology, Writing – original draft. LG: Writing – review & editing. YG: Writing – review & editing.
